# Progress Toward Achieving and Sustaining Maternal and Neonatal Tetanus Elimination — Worldwide, 2000–2022

**DOI:** 10.15585/mmwr.mm7328a1

**Published:** 2024-07-18

**Authors:** Camille E. Jones, Nasir Yusuf, Bilal Ahmed, Modibo Kassogue, Annemarie Wasley, Florence A. Kanu

**Affiliations:** ^1^Epidemic Intelligence Service, CDC; ^2^Global Immunization Division, Center for Global Health, CDC; ^3^Immunization, Vaccines and Biologicals, World Health Organization, Geneva, Switzerland; ^4^Maternal, Newborn, and Adolescent Health Program Division, UNICEF, New York, New York.

**Summary**

**What is already known about this topic?**

Tetanus causes considerable mortality among undervaccinated mothers and their infants following unhygienic deliveries, especially in low-income countries. The maternal and neonatal tetanus elimination initiative targets 59 priority countries.

**What is added by this report?**

During 2000–2022, 47 priority countries achieved maternal and neonatal tetanus elimination, contributing to global declines in neonatal tetanus cases (89%) and neonatal tetanus deaths (84%). Despite progress, the global disruption of routine immunization caused by the COVID-19 pandemic impeded elimination progress. Since 2020, reported neonatal tetanus cases have increased in 18 (31%) priority countries.

**What are the implications for public health practice?**

Integration of maternal and neonatal tetanus elimination strategies into priority countries’ national immunization activities is needed to achieve and sustain elimination globally.

## Abstract

Tetanus remains a considerable cause of mortality among undervaccinated mothers and their infants following unhygienic deliveries, especially in low-income countries. Strategies of the maternal and neonatal tetanus elimination (MNTE) initiative, which targets 59 priority countries, include strengthening antenatal immunization of pregnant women with tetanus toxoid–containing vaccines (TTCVs); conducting TTCV supplementary immunization activities among women of reproductive age in high-risk districts; optimizing access to skilled birth attendants to ensure clean deliveries and umbilical cord care practices; and identifying and investigating suspected neonatal tetanus cases. This report updates a previous report and describes progress toward MNTE during 2000–2022. By December 2022, 47 (80%) of 59 priority countries were validated to have achieved MNTE. In 2022, among the 50 countries that reported coverage with ≥2 doses of TTCV among pregnant women, 16 (32%) reported coverage of ≥80%. In 2022, among 47 validated countries, 26 (55%) reported that ≥70% of births were assisted by skilled birth attendants. Reported neonatal tetanus cases worldwide decreased 89%, from 17,935 in 2000 to 1,995 in 2021; estimated neonatal tetanus deaths decreased 84%, from 46,898 to 7,719. However, the global disruption of routine immunization caused by the COVID-19 pandemic impeded MNTE progress. Since 2020, reported neonatal tetanus cases have increased in 18 (31%) priority countries. Integration of MNTE strategies into priority countries’ national postpandemic immunization recovery activities is needed to achieve and sustain global elimination.

## Introduction

Maternal and neonatal tetanus* remains a substantial cause of mortality among undervaccinated mothers and their infants following unhygienic delivery, especially in low-income countries (*1*). In 1989, the World Health Assembly endorsed neonatal tetanus elimination.^†^ This activity was relaunched in 1999 as the maternal and neonatal tetanus elimination (MNTE)^§^ initiative, targeting 59 priority countries.^¶^ Because tetanus spores cannot be eliminated from the environment, and tetanus infection does not confer immunity, elimination requires ongoing active immunization with a tetanus toxoid–containing vaccine (TTCV). To protect infants from tetanus susceptibility at birth, women of reproductive age (usually 15–49 years) should be vaccinated with ≥2 doses of TTCV (TTCV2+), and immunization is recommended for undervaccinated pregnant women early in the third trimester (*2*). The MNTE initiative includes four strategies: 1) providing antenatal immunization of pregnant women with TTCV2+; 2) conducting TTCV supplementary immunization activities (SIAs)** in selected high-risk districts,^††^ targeting women of reproductive age for TTCV immunization; 3) supporting clean delivery and umbilical cord care practices through access to skilled birth attendants^§§^; and 4) identifying and investigating suspected neonatal tetanus cases with reliable surveillance (*2*,*3*). Since the MNTE initiative began in 1999, the estimated proportion of neonatal mortality attributed to tetanus decreased 84%, from 2% in 2000 to 0.3% in 2021.^¶¶^ The remaining risk for maternal and neonatal tetanus infection is concentrated in low-income communities with low TTCV coverage and limited access to hygienic delivery. This report summarizes progress toward achieving and sustaining MNTE during 2000–2022 and updates a previous report (*4*).

## Methods

### Immunization Activities, Deliveries by Skilled Birth Attendants, and Surveillance

To estimate TTCV coverage among pregnant women through routine immunization services and the number of neonates protected from tetanus at birth,*** the World Health Organization (WHO) and UNICEF use vaccination coverage survey data and administrative data^†††^ received from member countries (*5*). WHO and UNICEF also receive summaries of the number of women of reproductive age receiving TTCV during SIAs (*6*). The percentages of births assisted by skilled birth attendants are estimated from country health facility reports and coverage survey estimates (*7*). WHO recommends nationwide, case-based neonatal tetanus surveillance, active surveillance through regular visits to reporting sites (*8*), and country reports of neonatal tetanus case counts.^§§§^ Because most neonatal deaths occur in remote areas, which might lead to underreporting, neonatal tetanus deaths are estimated using mathematical models that project cause-specific neonatal mortality using Bayesian and multinomial frameworks (*9*). This activity was reviewed by CDC, deemed not research, and was conducted consistent with applicable federal law and CDC policy.^¶¶¶^

### Validation of Maternal and Neonatal Tetanus Elimination

Once a country’s surveillance data indicate that neonatal tetanus incidence has declined to <1 case per 1,000 live births in all districts, prevalidation assessments are conducted (*3*). Benchmarks for validating MNTE achievement include reaching <1 neonatal tetanus case per 1,000 live births, ≥80% routine TTCV2+ coverage among pregnant women, and ≥70% of deliveries assisted by skilled birth attendants. Assessments might also review supplementary measures, including TTCV2+ SIA coverage among women of reproductive age, antenatal care coverage,**** infant coverage with 3 doses of diphtheria, tetanus, and pertussis vaccine,^††††^ socioeconomic indices, field visits to determine health system performance, validation surveys in the poorest performing districts, and assessment of long-term plans for sustaining elimination.^§§§§^

### Maintenance of Maternal and Neonatal Tetanus Elimination

Once MNTE has been validated, WHO recommends that countries conduct annual neonatal tetanus risk analyses as part of immunization program reviews, and postvalidation assessments every 5 years, to determine whether elimination has been sustained and take any necessary corrective actions (*3*). The following indicators were used to determine maintenance of MNTE countries’ performance: 1) ≥80% TTCV2+ coverage among pregnant women accessing antenatal care, 2) ≥90% routine immunization TTCV coverage among children and adolescents (i.e., receipt of 3 primary infant doses and 3 booster doses), 3) ≥70% of deliveries by a skilled birth attendant, and 4) ≥90% of infants protected at birth against tetanus (*2*).

## Results

### Immunization Activities

**FIGURE 1:**
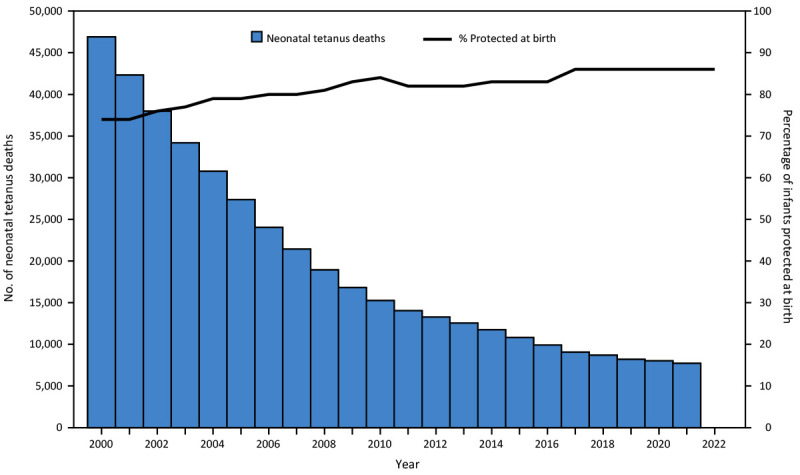
Estimated number of neonatal tetanus deaths*^,^^†^ and estimated percentage of infants protected at birth^§^^,^^¶^ against tetanus — worldwide, 2000–2022** **Abbreviations:** TTCV = tetanus toxoid-containing vaccine; WHO = World Health Organization. * The number of deaths is estimated from mathematical models that compute the yearly incidence and mortality for each country using the baseline rate of neonatal tetanus before TTCV introduction and promotion of clean deliveries, with adjustment for the estimated proportion of women vaccinated with TTCV and deliveries assisted by trained personnel. ^†^ Neonatal tetanus data from Child and Adolescent Cause of Death Estimation Group. ^§^ Protected at birth data from WHO/UNICEF Joint Reporting Form on Immunization (2000–2022). ^¶^ The status of an infant born to a mother who received 2 doses of TTCV during the last birth, ≥2 doses with the last dose received ≤3 years before the last delivery, ≥3 doses with the last dose received ≤5 years earlier, ≥4 doses with the last dose received ≤10 years earlier, or receipt of ≥5 previous doses. ** Death data for 2022 were not available.

**FIGURE 2:**
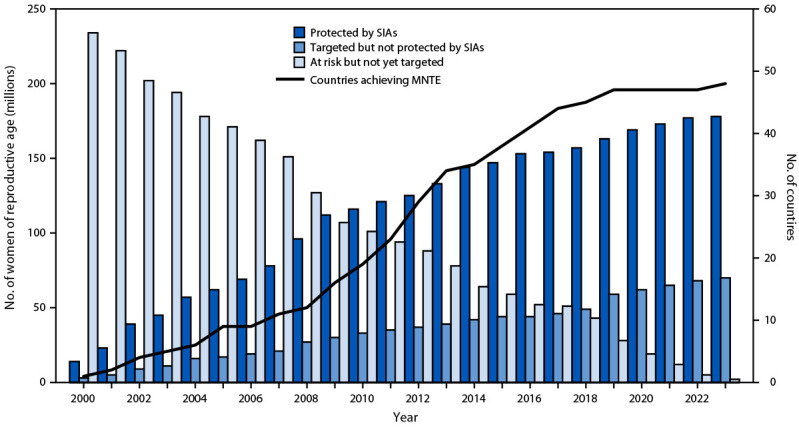
Number of women of reproductive age protected by tetanus toxoid–containing vaccine* received during supplementary immunization activities, number targeted^†^ but not yet vaccinated, number not yet targeted,^§^ and number of countries achieving maternal and neonatal tetanus elimination — 59 priority countries,^¶^ worldwide, 2000–2022 **Source:** WHO/UNICEF Maternal and Neonatal Tetanus Elimination Database, as of March 2024. **Abbreviations:** MNTE = maternal and neonatal tetanus elimination; SIAs = supplementary immunization activities; WHO = World Health Organization. * Protected with 2 doses of tetanus toxoid or tetanus and diphtheria toxoids. ^†^ Women of reproductive age included in SIA coverage goals. ^§^ Women of reproductive age estimated to be living in high-risk districts, which are yet to be targeted for tetanus toxoid–containing vaccine SIAs, primarily for programmatic reasons. ^¶^ Afghanistan, Angola, Bangladesh, Benin, Burkina Faso, Burma, Burundi, Cambodia, Cameroon, Central African Republic, Chad, China, Comoros, Congo, Côte d’Ivoire, Democratic Republic of the Congo, Egypt, Equatorial Guinea, Eritrea, Ethiopia, Gabon, Ghana, Guinea, Guinea-Bissau, Haiti, India, Indonesia, Iraq, Kenya, Laos, Liberia, Madagascar, Malawi, Mali, Mauritania, Mozambique, Namibia, Nepal, Niger, Nigeria, Pakistan, Papua New Guinea, Philippines, Rwanda, Senegal, Sierra Leone, Somalia, South Africa, South Sudan, Sudan, Timor-Leste, Togo, Turkey, Uganda, Tanzania, Vietnam, Yemen, Zambia, and Zimbabwe.

In 2022, among 59 priority countries, 50 (85%) reported antenatal TTCV2+ coverage data; 16 (32%) of these reported ≥80% TTCV2+ coverage. During 2000–2022, a total of 52 (88%) priority countries conducted TTCV SIAs (Table). Among 41 countries with 2000 and 2022 data available, TTCV2+ coverage increased in 30 (73%). Worldwide, the proportion of infants protected at birth increased from 74% in 2000 to 86% in 2022 (Figure 1), and the number of priority countries that achieved MNTE increased from 1 (2%) of 57 in 2000 to 47 (80%) of 59 in 2022 (Figure 2).

**TABLE:** Indicators of achievement of maternal and neonatal tetanus elimination — 59 priority countries,* 2000–2022

Country	Year of MNTE validation	≥2 TTCV doses among pregnant women, %^†,§^			Newborns protected at birth, %^¶^			Women of reproductive age vaccinated during TTCV SIAs**		Skilled birth attendant at delivery, %^††^			No. of neonatal tetanus cases^§§^		
		2000	2022	% Change 2000–2022	2000	2022	% Change 2000–2022	No. of TT2+/Td2 doses administered	% Vaccinated	2000^¶¶^	2022^¶¶^	% Change 2000–2022	2000	2022	% Change 2000–2022
Bangladesh***	2008	89	47	−48	89	98	10	1,438,374	47	12	59	388	376	19	–95
Benin***	2010	81	89	10	87	83	−5	1,399,461	97	66	78	19	52	18	–65
Burkina Faso^†††^	2012	NA	93	NA	57	95	67	2,306,835	91	38	96	154	22	1	–95
Burma	2010	81	55	−32	79	88	11	8,170,763	87	57	NA	NA	41	14	–66
Burundi	2009	28	60	114	51	87	71	679,222	55	25	77	204	16	0	–100
Cambodia	2015	40	69	72	58	93	60	2,099,471	79	32	99	210	295	7	–98
Cameroon	2012	40	54	35	54	81	50	2,687,461	85	56	69	23	279	40	–86
Chad	2019	12	84	598	39	75	92	3,222,840	84	14	47	245	142	269	89
China	2012	NA	NA	NA	NA	NA	NA	NA	NA	97	NA	NA	3,230	18	–99
Comoros	2009	40	78	96	57	83	46	160,767	55	62	NA	NA	NA	2	NA
Côte d'Ivoire	2013	78	72	−8	76	83	9	5,924,527	85	63	84	34	30	27	–10
Democratic Republic of the Congo	2019	25	96	284	45	80	78	10,342,937	92	61	85	40	77	15	–81
Egypt	2007	71	97	37	80	88	10	2,518,802	87	61	97	59	321	1	–100
Equatorial Guinea	2016	30	21	−30	61	60	−2	26,466	9	65	NA	NA	NA	0	NA
Eritrea	2003	25	65	160	80	99	24	NA	NA	28	NA	NA	4	0	–100
Ethiopia	2017	32	NA	NA	54	85	57	13,210,107	84	6	50	789	20	NA	NA
Gabon^†††^	2013	16	43	171	39	83	113	79,343	90	86	95	11	8	8	0
Ghana	2011	73	63	−13	69	90	30	1,666,666	87	47	79	68	80	0	–100
Guinea-Bissau***	2012	NA	30	NA	49	80	63	312,669	98	32	54	69	NA	3	NA
Haiti	2017	NA	37	NA	41	78	90	2,785,588	88	24	42	75	40	NA	NA
India	2015	80	85	7	85	93	9	7,643,440	94	43	89	110	3,287	65	–98
Indonesia	2016	81	70	−14	82	83	1	1,442,264	50	66	95	43	466	21	–95
Iraq	2013	55	NA	NA	75	73	−3	111,721	96	65	96	47	37	NA	NA
Kenya^†††^	2018	51	65	27	68	85	25	4,463,695	67	43	89	109	1,278	NA	NA
Laos	2013	45	5	−89	58	93	60	968,323	90	17	64	286	21	NA	NA
Liberia	2011	25	64	156	51	90	76	288,984	57	51	84	66	152	12	–92
Madagascar	2014	40	51	27	58	75	29	2,705,588	72	47	46	–3	13	19	46
Malawi	2002	61	1	−98	84	90	7	NA	NA	56	96	73	12	5	–58
Mali^§§§^	2023	62	70	13	50	83	66	4,158,201	49	41	67	66	73	3	–96
Mauritania	2015	NA	29	NA	44	81	84	586,277	76	53	70	32	NA	0	NA
Mozambique^†††^	2010	61	NA	NA	75	84	12	605,640	79	48	68	42	42	105	150
Namibia	2001	60	36	−41	74	90	22	NA	NA	76	NA	NA	10	0	–100
Nepal	2005	60	93	55	67	91	36	4,537,864	86	12	77	549	134	3	–98
Niger	2016	31	80	158	63	83	32	2,184,277	92	16	44	178	55	20	–64
Philippines	2017	58	NA	NA	55	91	65	1,034,080	78	58	84	46	281	54	–81
Republic of the Congo	2009	39	83	114	67	87	30	273,003	91	83	91	9	2	8	300
Rwanda***	2004	NA	76	NA	81	97	20	NA	NA	31	94	201	5	7	40
Senegal	2011	45	100	123	62	96	55	359,845	92	58	75	29	0	1	NA
Sierra Leone	2013	20	84	320	53	93	75	1,704,814	102	37	87	134	36	5	–86
South Africa	2002	65	NA	NA	68	88	29	NA	NA	91	NA	NA	11	0	–100
Tanzania	2012	77	90	17	79	90	14	987,575	NA	43	64	46	48	13	–73
Timor-Leste	2012	NA	34	NA	NA	85	NA	24,141	53	24	NA	NA	NA	1	NA
Togo	2005	47	74	58	63	83	32	262,130	87	35	69	96	33	12	–64
Turkey	2009	36	68	89	50	97	94	1,242,674	58	83	97	17	26	0	–100
Uganda	2011	42	59	41	70	81	16	2,448,527	86	39	NA	NA	470	NA	NA
Vietnam***	2005	90	88	−2	86	96	12	367,842	69	59	96	63	142	33	–77
Zambia	2007	61	NA	NA	78	83	6	330,030	81	42	80	91	130	50	–62
Zimbabwe	2000	60	NA	NA	76	89	17	NA	NA	NA	86	NA	16	0	–100
**Maternal and neonatal tetanus elimination not validated by the end of 2023**															
Afghanistan	—	20	92	361	32	60	88	5,212,394	45	14	62	332	139	20	–86
Angola	—	NA	39	NA	60	65	8	7,097,552	84	NA	50	NA	131	239	82
Central African Republic	—	20	84	320	36	65	81	2,595,415	42	32	40	27	37	38	3
Guinea	—	43	90	109	79	80	1	4,957,272	49	49	55	14	245	85	–65
Nigeria***	—	NA	43	NA	57	67	18	13,820,506	51	35	51	44	1,643	55	–97
Pakistan	—	51	66	29	71	86	21	28,219,661	81	23	68	196	1,380	509	–63
Papua New Guinea***	—	10	36	256	24	65	171	450,739	15	39	56	45	138	13	–91
Somalia	—	22	74	234	47	57	21	497,561	27	19	32	65	966	0	–100
South Sudan	—	NA	51	NA	NA	65	NA	6,247,983	56	NA	40	NA	NA	0	NA
Sudan	—	34	NA	NA	61	81	33	7,365,615	86	NA	NA	NA	88	NA	NA
Yemen	—	31	24	−23	54	73	35	3,612,931	51	27	NA	NA	174	132	−24

**Abbreviations**: MNTE = maternal and neonatal tetanus elimination; NA = not available; SIA = supplementary immunization activity; TT2+ and Td2+ = ≥2 doses of tetanus toxoid and tetanus-diphtheria toxoid; TTCV = tetanus toxoid–containing vaccine; WHO = World Health Organization.

* Afghanistan, Angola, Bangladesh, Benin, Burkina Faso, Burma, Burundi, Cambodia, Cameroon, Central African Republic, Chad, China, Comoros, Côte d’Ivoire, Democratic Republic of the Congo, Egypt, Equatorial Guinea, Eritrea, Ethiopia, Gabon, Ghana, Guinea, Guinea-Bissau, Haiti, India, Indonesia, Iraq, Kenya, Laos, Liberia, Madagascar, Malawi, Mali, Mauritania, Mozambique, Namibia, Nepal, Niger, Nigeria, Pakistan, Papua New Guinea, Philippines, Republic of the Congo, Rwanda, Senegal, Sierra Leone, Somalia, South Africa, South Sudan, Sudan, Tanzania, Timor-Leste, Togo, Turkey, Uganda, Vietnam, Yemen, Zambia, and Zimbabwe.

^†^ TTCV data from WHO/UNICEF Joint Reporting Form on Immunization (2000–2022).

^§^ Includes first-year SIA conducted in Bangladesh in 1999 and first- and second-year SIAs conducted in Ethiopia in 1999.

^¶^ Protected at birth data from WHO/UNICEF Joint Reporting Form on Immunization (2000–2022).

** SIA data from WHO/UNICEF MNTE Database, as of March 2024.

^††^ Skilled birth attendant data from WHO Global Health Observatory Data Repository (2000–2022).

^§§^ Neonatal tetanus case data from WHO Global Health Observatory Data Repository (2000–2022).

^¶¶^ Includes skilled birth attendant surveys conducted within 5 years for years 2000 and 2022.

*** Administrative ≥2-dose TTCV coverage among women of reproductive age was used when official data were unavailable for selected country.

^††† ^Skilled birth attendant data were extracted from country-specific demographic health surveys. https://www.dhsprogram.com/data/available-datasets.cfm

^§§§^ MNTE was not validated in Mali by the end of 2022; however, elimination was validated in 2023.

During 2000–2022, SIAs provided TTCV2+ to 177 million (70%) of 252 million women of reproductive age targeted to receive vaccination. During 2021–2022, seven countries conducted TTCV SIAs, vaccinating 13 million women of reproductive age. However, by the end of 2022, 68 million women who were targeted for protection by TTCV SIAs remained unreached.

### Deliveries Assisted by Skilled Birth Attendants

In 2022, among 47 priority countries with available data, 26 (55%) reported that ≥70% of births were assisted by skilled birth attendants (Table). Compared with the most recent report (*4*), the proportions of births assisted by skilled birth attendants was higher in 12 countries (Afghanistan, Burkina Faso, Cambodia, Chad, Côte d’Ivoire, Egypt, Kenya, India, Malawi, Mauritania, Niger, and Nigeria) in 2022 than in 2020.

### Neonatal Tetanus Surveillance and Incidence

Among the 59 MNTE priority countries, 11 (19%) reported zero neonatal tetanus cases in 2022; however, seven countries reported more cases in 2022 than in 2000 (Table). Worldwide, reported neonatal tetanus cases decreased by 89%, from 17,935 in 2000 to 1,995 in 2021. Estimated neonatal tetanus deaths decreased 84%, from 46,898 in 2000 to 7,719 in 2021, accounting for 2% and 0.3% of all-cause neonatal mortality, respectively (Figure 1). Since 2020, reported neonatal tetanus cases have increased in 18 (31%) priority countries, including 13 previously validated countries.

### Validation of Maternal and Neonatal Tetanus Elimination

During 2000–2022, 47 (80%) of the 59 priority countries were validated to have achieved MNTE (Table). No countries achieved validation during 2020–2022; however, MNTE was validated in Mali in 2023.

### Maintenance of Maternal and Neonatal Tetanus Elimination

As of 2022, among 47 MNTE-validated countries, 15 (32%) achieved ≥90% coverage with 3 primary doses of routine immunization TTCV. TTCV booster doses were included in the routine immunization schedule for children aged 12–23 months in 14 (30%) of those countries, and for children and adolescents aged 4–7 and 9–15 years in 11 (23%) countries. In 46 (98%) countries, ≥70% of infants were protected at birth against tetanus; and in 26 (55%), ≥70% of births were assisted by a skilled birth attendant (Table). Six countries have conducted postvalidation assessments.

## Discussion

Substantial progress has been made toward global MNTE, with 80% of priority countries validated as having achieved elimination by the end of 2022. TTCV2+ coverage increased in 30 priority countries, and in 26 countries, skilled birth attendants assisted in ≥70% of births. Since 2000, 52 priority countries have conducted TTCV SIAs. During 2021–2022, seven countries yet to achieve MNTE conducted SIAs, reaching 13 million (42%) women of reproductive age with TTCV2+ and contributing to a 16% increase in the number of infants protected at birth. Worldwide, during 2000–2022, the number of reported neonatal tetanus cases declined by 89%, from 17,935 to 1,995, and estimated neonatal tetanus mortality decreased 84%, from 46,898 to 7,719, since 2000. In addition, by 2022 four of six geopolitical zones in Nigeria, and Punjab province in Pakistan were validated to have achieved elimination.^¶¶¶¶^

Although progress has been substantial, challenges to MNTE remain, some of which were amplified by the COVID-19 pandemic and its global disruption of immunization services.***** Many countries that have not yet validated MNTE have fragile health systems with barriers to improving vaccination coverage and accessing skilled birth attendants. For example, in countries experiencing political instability and conflict, more areas might be hard to reach, magnifying the challenges to providing immunization and safe hygienic deliveries, as well as ensuring reliable detection of and response to occurrent neonatal tetanus cases. Recovery of national immunization programs has been challenging in some countries that experienced increases in some vaccine-preventable diseases in the wake of the COVID-19 pandemic.

In addition to continuing measures to achieve global MNTE, more attention is needed to ensure that elimination is sustained in countries previously validated to have achieved MNTE. Since 2020, reported neonatal tetanus cases have increased in 13 previously validated countries. This increase might indicate better surveillance system sensitivity; however, it might also reflect lack of protection at birth and the need for improved antenatal vaccination measures. By 2022, only one third of 43 MNTE–validated countries sustained ≥80% TTCV2+ coverage, and in 12 MNTE-validated countries, fewer than 70% of births were assisted by skilled birth attendants. As of 2022, fewer than one third of validated countries had introduced ≥1 TTCV booster dose into their routine immunization schedule. This slow introduction might be attributed to lower prioritization of MNTE activities after validation because of funding constraints, putting countries at risk for reemergence of neonatal tetanus (*3*).

Sustaining MNTE requires strong commitments from priority countries and the global community. Countries will need to improve resource and program efficiency by integrating postvalidation assessments with immunization program reviews and TTCV booster dose vaccination with other immunization activities (e.g., school vaccination programs). Innovative activities to integrate neonatal tetanus case-based surveillance into surveillance for other vaccine-preventable diseases, such as polio and measles, might support system efficiency and sustainability, and public engagement might help raise awareness and strengthen community-based vaccine-preventable disease surveillance systems (*8*).

### Limitations

The findings in this report are subject to at least three limitations. First, reported TTCV2+ coverage among pregnant women can underestimate actual protection because it does not account for women who received TTCV doses in previous pregnancies but were unvaccinated during their current pregnancy (*2*). Second, whereas MNTE validation is based on district-level assessments, reports of immunization coverage used in this update are based on national estimates and might obscure interdistrict differences. Finally, neonatal deaths are estimated using mathematical models (*9*); thus, estimates are subject to model assumptions.

### Implications for Public Health Practice

MNTE has been included in the WHO Immunization Agenda 2030^†††††^ global strategy as an endorsed vaccine-preventable disease elimination target. As part of the worldwide effort to increase immunization coverage after the COVID-19 pandemic, integration of MNTE activities with those of other vaccine-preventable diseases is needed to improve progress toward MNTE. One such strategy includes promoting a life course approach to vaccination by integrating TTCV booster doses in school health programs and in other life course immunization platforms (*10*). Promotion of equitable access to health services, such as clean deliveries, is also important to achieving MNTE.

## Footnotes

* Tetanus is an infection caused by *Clostridium tetani,* a bacterium that produces a potent toxin. Maternal tetanus occurs during pregnancy or within 6 weeks of the end of pregnancy and might occur during abortion, miscarriage, or birth with unhygienic delivery. Neonatal tetanus occurs during the first 28 days of life, after either the cutting of the umbilical cord under nonsterile conditions or applying nonsterile traditional remedies to the umbilical stump in an infant who does not have passively (transplacentally) acquired maternal antibodies (i.e., the mother is not immune to tetanus).

† The occurrence of <1 neonatal tetanus case per 1,000 live births per year in every district in every country.

§ Neonatal tetanus elimination is considered a proxy for maternal tetanus elimination; the same strategies for elimination are common to both.

¶ Afghanistan, Angola, Bangladesh, Benin, Burkina Faso, Burma, Burundi, Cambodia, Cameroon, Central African Republic, Chad, China, Comoros, Republic of the Congo, Côte d'Ivoire, Democratic Republic of the Congo, Egypt, Equatorial Guinea, Eritrea, Ethiopia, Gabon, Ghana, Guinea, Guinea-Bissau, Haiti, India, Indonesia, Iraq, Kenya, Laos, Liberia, Madagascar, Malawi, Mali, Mauritania, Mozambique, Namibia, Nepal, Niger, Nigeria, Pakistan, Papua New Guinea, Philippines, Rwanda, Senegal, Sierra Leone, Somalia, South Africa, Sudan, Togo, Turkey, Uganda, Tanzania, Vietnam, Yemen, Zambia, and Zimbabwe. The creation of Timor-Leste in 2002 and South Sudan in 2011 increased the number of priority countries to 59.

** Mass vaccination campaigns conducted in three rounds to administer 3 doses of TTCV to women of reproductive age in high-risk districts.

†† Districts considered to be at high risk because the estimated neonatal tetanus case rate exceeds one per 1,000 live births, clean delivery coverage is <70%, and 3-dose TTCV coverage among pregnant women during the previous 5 years is <80%.

§§ A doctor, nurse, midwife, or health worker trained in providing lifesaving obstetric care, including giving necessary supervision, care, and advice to women during pregnancy, childbirth, and the postpartum period.

¶¶ Neonatal mortality data were unavailable for 2022. https://platform.who.int/data/maternal-newborn-child-adolescent-ageing/indicator-explorer-new/mca/number-of-neonatal-deaths---by-cause

*** The status of an infant born to a mother who received 2 doses of TTCV during the previous birth, ≥2 doses with the last dose received ≤3 years before the last delivery, ≥3 doses with the last dose received ≤5 years earlier, ≥4 doses with the last dose received ≤10 years earlier, or receipt of ≥5 previous doses.

††† Administrative data to calculate the number of neonates protected at birth estimates the number of doses administered through routine services (numerator) divided by the number in target group (denominator) × 100.

§§§
https://www.who.int/data/gho/data/indicators/indicator-details/GHO/neonatal-tetanus---number-of-reported-cases

¶¶¶ 45 C.F.R. part 46.102(l)(3), 21 C.F.R. part 56; 42 U.S.C. Sect. 241(d); 5 U.S.C. Sect. 552a; 44 U.S.C. Sect. 3501 et seq.

**** Antenatal care coverage is the percentage of females aged 15–49 years with a live birth who received antenatal care provided by a skilled birth attendant at least once during pregnancy.

††††
https://www.who.int/data/gho/data/indicators/indicator-details/GHO/diphtheria-tetanus-toxoid-and-pertussis-(dtp3)-immunization-coverage-among-1-year-olds-(-)

§§§§
https://cdn.who.int/media/docs/default-source/immunization/mnte/who-ivb-18.15-eng.pdf

¶¶¶¶
https://www.who.int/initiatives/maternal-and-neonatal-tetanus-elimination-(mnte)/the-partnership

*****
https://www.who.int/news/item/15-07-2021-covid-19-pandemic-leads-to-major-backsliding-on-childhood-vaccinations-new-who-unicef-data-shows

†††††
https://www.who.int/teams/immunization-vaccines-and-biologicals/strategies/ia2030
